# Rapid detection of carbapenem resistance: targeting a zero level of inadequate empiric antibiotic exposure?

**DOI:** 10.1186/s13054-016-1582-0

**Published:** 2016-12-19

**Authors:** Andrea Cortegiani, Vincenzo Russotto, Pasquale Iozzo, Santi Maurizio Raineri, Antonino Giarratano

**Affiliations:** 1 Department of Biopathology and Medical Biotechnologies (DIBIMED), Section of Anaesthesia, Analgesia, Intensive Care and Emergency, University Hospital Paolo Giaccone, University of Palermo, Via del vespro 129, 90127 Palermo, Italy; 2 Intensive Care Unit, University Hospital Paolo Giaccone, University of Palermo, Via del vespro 129, 90127 Palermo, Italy

**Keywords:** Multidrug-resistant bacteria, Carbapenem resistance, Polymerase chain reaction

Resistance to carbapenems is an increasingly encountered phenomenon in the ICU, complicating empiric and targeted antimicrobial therapy. Infections due to carbapenem-resistant microorganisms are characterized by high morbidity and mortality [1, 2]. Recently, there has been an increasing interest in rapid detection techniques, based on real time on-demand easy-to-use PCR, to detect genes responsible for carbapenem resistance. One of these techniques is the Cepheid Xpert Carba-R assay, which is able to detect and differentiate five of the most frequent genes associated with non-susceptibility to carbapenems in Gram-negative bacteria (*bla*_*KPC*_, *bla*_*VIM*_, *bla*_*OXA-48*_, *bla*_*IMP-1*_, *bla*_*NDM*_). The diagnostic performance of this assay seems to be high when compared to classic microbiological cultures and gene identification with in-house PCR in a clinical setting, especially in intra-abdominal infections using samples from rectum or abdominal drainage material [3, 4]. Originally, assays for screening of patients carrying multidrug-resistant organisms were used to guide infection control programs, to restrict access to patients’ health-care zones, or for outbreak surveillance. However, several studies reported an association between detection from surveillance techniques and subsequent infection etiology, improving the rate of adequate empiric antimicrobial treatment [5].

The Xpert Carba-R assay delivers results in less than 1 hour. It might be argued that the identification of genes associated with non-susceptibility at the beginning of therapeutic decision-making, when clinicians are deciding whether to add carbapenems or not, may lower the rate of inadequate use of these antimicrobials, to which resistance rates are continuously increasing. This application would shift the utility of this technique from screening to an active part of therapeutic decision-making. To date, two aspects may limit its application. First, although it is able to detect five of the most common genes, clinicians should carefully evaluate which are the genetic determinants of carbapenem-resistance in their ICU to optimize diagnostic performance. Second, the data came from rectal swabs and material from abdominal drainage and the clinical usefulness in other clinical settings should be tested.

Following this road, we may hope to obtain, in the near future, information on genes associated with resistance to antibiotics by easy-to-use, on-demand techniques before starting therapies and reducing inadequate empiric treatment to nearly zero.

## Abbreviations

PCR: Polymerase chain reaction
